# Integration of text mining and biological network analysis: Identification of essential genes in sulfate-reducing bacteria

**DOI:** 10.3389/fmicb.2023.1086021

**Published:** 2023-04-13

**Authors:** Priya Saxena, Shailabh Rauniyar, Payal Thakur, Ram Nageena Singh, Alain Bomgni, Mathew O. Alaba, Abhilash Kumar Tripathi, Etienne Z. Gnimpieba, Carol Lushbough, Rajesh Kumar Sani

**Affiliations:** ^1^Department of Chemical and Biological Engineering, South Dakota School of Mines and Technology, Rapid City, SD, United States; ^2^Data Driven Material Discovery Center for Bioengineering Innovation, South Dakota School of Mines and Technology, Rapid City, SD, United States; ^3^2-Dimensional Materials for Biofilm Engineering, Science and Technology, South Dakota School of Mines and Technology, Rapid City, SD, United States; ^4^Department of Biomedical Engineering, University of South Dakota, Sioux Falls, SD, United States; ^5^BuG ReMeDEE Consortium, South Dakota School of Mines and Technology, Rapid City, SD, United States

**Keywords:** *Oleidesulfovibrio alaskensis* G20, essential genes, pathways, semi-automated model, SRB, text mining

## Abstract

The growth and survival of an organism in a particular environment is highly depends on the certain indispensable genes, termed as essential genes. Sulfate-reducing bacteria (SRB) are obligate anaerobes which thrives on sulfate reduction for its energy requirements. The present study used *Oleidesulfovibrio alaskensis* G20 (OA G20) as a model SRB to categorize the essential genes based on their key metabolic pathways. Herein, we reported a feedback loop framework for gene of interest discovery, from bio-problem to gene set of interest, leveraging expert annotation with computational prediction. Defined bio-problem was applied to retrieve the genes of SRB from literature databases (PubMed, and PubMed Central) and annotated them to the genome of OA G20. Retrieved gene list was further used to enrich protein–protein interaction and was corroborated to the pangenome analysis, to categorize the enriched gene sets and the respective pathways under essential and non-essential. Interestingly, the *sat* gene (dde_2265) from the sulfur metabolism was the bridging gene between all the enriched pathways. Gene clusters involved in essential pathways were linked with the genes from seleno-compound metabolism, amino acid metabolism, secondary metabolite synthesis, and cofactor biosynthesis. Furthermore, pangenome analysis demonstrated the gene distribution, where 69.83% of the 116 enriched genes were mapped under “persistent,” inferring the essentiality of these genes. Likewise, 21.55% of the enriched genes, which involves specially the formate dehydrogenases and metallic hydrogenases, appeared under “shell.” Our methodology suggested that semi-automated text mining and network analysis may play a crucial role in deciphering the previously unexplored genes and key mechanisms which can help to generate a baseline prior to perform any experimental studies.

## 1. Introduction

Essential genes are the foundation of life due to their decisive role in survival and development of an organism (Peng et al., 2017). These genes play crucial role in deciphering the essential survival mechanism and key cellular functions in any living organism (Rajeev et al., 2011). Regulation of such essential genes are majorly controlled by the “Central Dogma” process and any deletion or mutation of such genes can adversely impact the survival of the organism (Keskin et al., 2016). The term “essential gene” is highly context-dependent (Rancati et al., 2018). It can be defined as the “minimal gene set” required for a cell to carry out basic metabolism and reproduction under optimal and ambient conditions (Lachance et al., 2021). Essential genes tend to be highly conserved and are involved in regulating essential cellular processes such as replication, transcription, and translation (Keskin et al., 2016). Besides, the genes associated with these processes are highly conserved and the pathways (e.g., nucleotide metabolism and amino acid synthesis) associated with them are essential to carry out basic metabolism required for the growth and survival of an organism (Gil et al., 2004). Based on central dogma and sulfate respiration system, we have categorized the essential genes in the functional categories of Carbon and energy metabolism, Nucleotide metabolism, Ribosome synthesis, Amino acid synthesis, Two-component signaling system and Sulfur metabolism (Saxena et al., 2021).

The identification of essential genes and their role in critical cellular processes are of great interest in the field of evolutionary microbiology and genetics (Dong et al., 2020). Generally, two approaches are most frequently used to determine essential genes: (a) experimental and (b) computational methods (Juhas et al., 2011). Experimental methods include molecular techniques such as transposon mutagenesis and CRISPR cas9 based and multi-omics based strategies (Razzaq et al., 2021). These methods are time consuming and expensive, moreover, different experimental methods may generate distinct results which require a number of cross-validation studies (Pinu et al., 2019). On other hand, *in silico* approach to find the key essential genes came in scenario as early as 1996, where Mushegian and Koonin assessed genomes of *Haemophilus influenzae* and *Mycoplasma genitalium* to ascertain the minimal gene set (Xavier et al., 2014). In addition, the rapid advancement in sequencing technology has enhanced the reliability of computational methods in identification of bacterial gene essentiality (Merwin et al., 2020; Nandi et al., 2020). Retrieval of data is the most critical step in any computational method applied towards identifying essential genes (Brynjolfsson and Hitt, 1998). The key basis of any computational study involves the development of an algorithm-based program using text mining and predictive modeling approaches (Rosário-Ferreira et al., 2021). The text mining algorithm is used to extract data from relevant literature in different databases such as database of essential genes, online gene essentiality database, essential genes of genome scale, and cluster essential genes (Nembot et al., 2021). However, one of the major limitations of text mining *via* machine learning algorithms includes their inability to identify the essential genes on the condition basis due to the unavailability of classified data to train the text mining algorithm (Aromolaran et al., 2021). Moreover, the sufficient gene corpus collection with the weighted and unweighted scoring of genes to predict its essentiality varies significantly among the databases (Li et al., 2020). Moving forward, the community engagement to generate more accurate corpora and generalized transformer models such as ChatGPT implementation may address this completeness when enough data are generated in specific community. To circumvent these limitations, an alternative and simplified analysis of the genes using pangenome analysis can be assessed to predict the essentiality of the genes. Pangenome is the collection of entire set of orthologous genes for a group of genomes (Ding et al., 2018). Pangenome analysis can be used to decipher the dispensable genome, i.e., the sequence of gene set conserved across all the individual species in a genera (Koonin, 2002). Some worldwide applications using pangenome includes determining the target for vaccine development, helps in classification of microorganism in taxonomic unit, to understand the evolution of pathogenic species by the identification of virulence gene shared among all the pathogenic species (Costa et al., 2020). Therefore, pangenome analysis would be a crucial and significant addition to the existing text mining strategies to obtain a strong baseline for gene essentiality (Koonin, 2002; Tettelin et al., 2005).

Sulfate reducing bacteria (SRB) are obligate anaerobes that use sulfate as a source of electron acceptor and reduce it to hydrogen sulfide (Krayzelova et al., 2015). Despite of relentless increase in the number of published articles that belong to diverse research areas from industrial biotechnology (e.g., removal of heavy metals and waste valorization) to molecular biology (e.g., genetic architecture of the genes in biocorrosion and biofilm formation on metal surfaces), not much information about the essential genes of SRB community is known yet. Therefore, research on essential genes, is quite appealing to identify the potential genes, which can substantially help in deciphering the survival mechanisms of SRB. The *Oleidesulfovibrio alaskensis* G20 (OA G20; earlier *Desulfovibrio alaskensis* G20) is a SRB known to corrode metals and cause souring of petroleum. The survival mechanism of OA G20 under extreme conditions (e.g., high metal active environment) can be deciphered through identification of essential genes. However, till date a large number of proteins (≈577 proteins) are uncharacterized in OA G20. So far, no categorization is available for the genes of OA G20 with reference to essentiality. In this study, manual and semi-automated text mining approach was accessed to retrieve the genes of SRB from different literature databases. In addition, the annotation and mapping of the genes from different SRB into the genome of OA G20 were performed. With the conjoint study of physical protein–protein interactions and pangenome analysis, this article elucidated an interesting approach to classify genes under the category essential and non-essential. Finally, this study listed and detailed the essential genes and pathways of OA G20 and their principle biochemical mechanism that assist the cell survivability.

## 2. Materials and methods

### 2.1. Data mining and data subsets

To accomplish the objective of essential genes, initially, articles were retrieved from literature databases- PubMed and PubMed Central (PMC), using an automated definite query with keywords– “sulfate reducer,” “SRB,” “Sulfate reducing bacteria,” “G20,” “Essential Gene,” and “Gene essential.” Relevant peer-reviewed and non-peer-reviewed articles on the essential genes of SRB were compiled, screened, and analyzed. Manual curation on the hits was performed by examining profoundly the contents of the papers, and appropriate articles that contain the SRB genes and were screened down. A manageable set of genes were manually mined from the screened articles, and concurrently a python based automated text mining approach was used to extract genes from the articles. This automated text mining tool is a NER (Name Entity Recognition), which leverages a trained machine learning model to automatically retrieve genes on various targeted papers. The machine learning model is trained on pre-annotated dataset using our recently highly accurate (accuracy up to 97%) and published framework (Fotseu et al., 2021). In the gene annotation process, genes are highlighted for datasets preparation to train a model that can effectively recognize the gene. Subsequently, elimination of the genes that were not evolutionary homologous to the *Desulfovibrio* genus, and the genes that were identical, further reduced the number of starting genes.

### 2.2. Computational and machine learning pipeline

#### 2.2.1. Text mining and NER workflow

**Figure 1:**
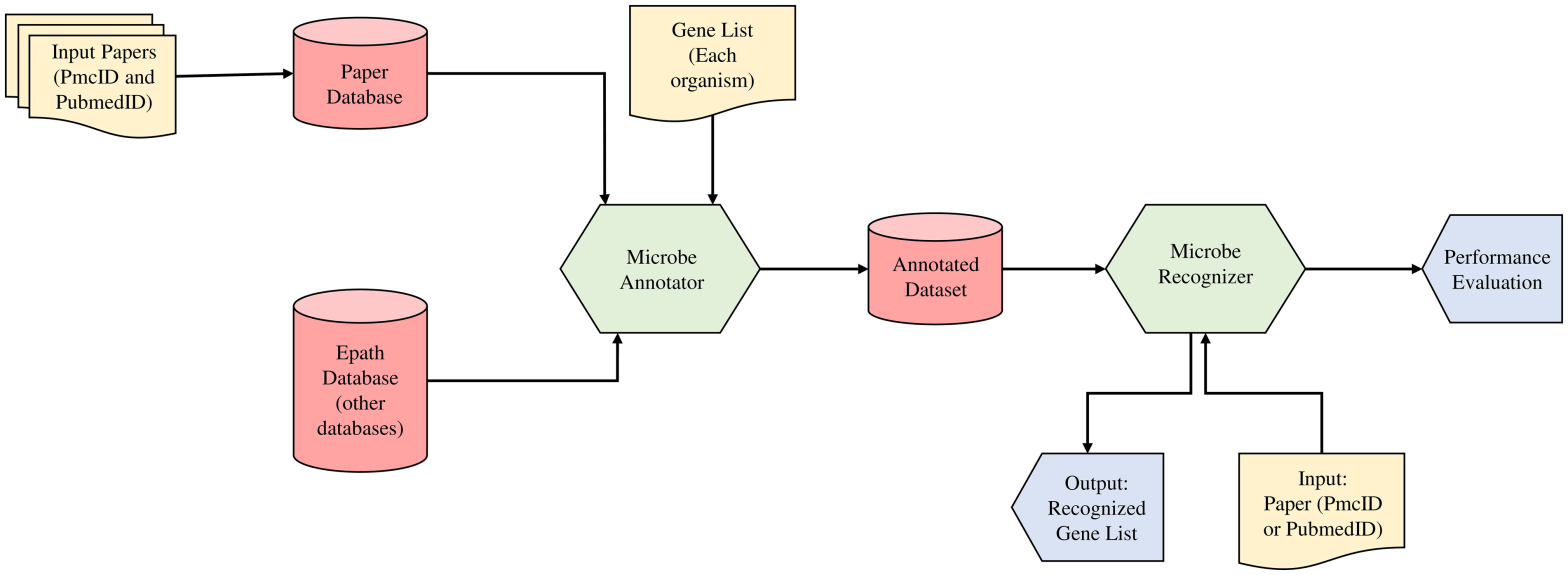
The text mining method based on Name Entity Recognition principle (NER) state of the art machine learning model. The study incorporated two modules (Microbe annotator and Microbe recognizer) in the predictive model for the gene prediction and feature analysis from literature databases and EPATH databases, respectively.

Despite the growing number of gene recognition algorithms and models, there is a limited customizable model for microorganism gene recognition. For example, the NCBI NER tool named Pubtator is unable to recognize G20 genes from this paper: PMC1913334 (Wei et al., 2019; Fotseu et al., 2021). To resolve this issue in our workflow, we proposed as shown in Figure 1, a text mining method based on NER principle state of the art machine learning model (Fotseu et al., 2021). Our workflow includes two modules, the “Microbe annotator” and the “Microbe Recognizer” modules to annotate the dataset corpus that will be used to train our recognizer model.

#### 2.2.2. Microbe annotator

Advances in the biomedical field today have made it possible to have a multitude of research works indexed in several online databases. With the growing amount of information available online, one of the biggest challenges is managing unstructured data and making it machine-readable. This is the role of the text annotation. Microbe annotator is an organism specific gene annotation module that will take as input the full list of every gene in a given organism and transform it into a dictionary. The dictionary is then used to create a corpus that will contain all genes relevant to our organism of interest (here G20 organism). The customization of the dataset preparation is done by getting as input the basic and important paper collection set related to user problem in publication database (PubMed, and PMC), the gene list of the targeted organism G20 from online databases KEGG and EPATH. Then as output of this module, we have an annotated dataset. A set of paper with annotated genes located at different positions in the free text format. This custom corpus is then used to train the recognizer.

#### 2.2.3. Microbe recognizer

This module aims to build a text mining model that will recognize any gene relevant to a specific problem of interest (here G20 gene collection). The recognizer prepares the annotated dataset, trains, evaluates and tests our model on it. During the process, the data is transformed from the original PMC and PubMed XML format into the JSONL format (input format of the second module). The dataset follows three sub-steps: granularization, pre-processing, training, evaluation and test. Granularization makes it possible to manage too-long text, automatically generate new annotations and extract special characters. For model training, the different dataset is subdivided into several slices and performs continuous training of the model. Subsequently, an evaluation of the model is made. The aim is to make the final model recognize every possible gene in our organism of interest, even if no abstract or paper contains all of them. The model is then tested on the subset of our data that wasn’t used for training. Simply pass the PMCID or PubMed ID of a paper as input to obtain the gene list recognized by the module in the paper as output.

#### 2.2.4. Essential gene prediction workflow

**Figure 2:**
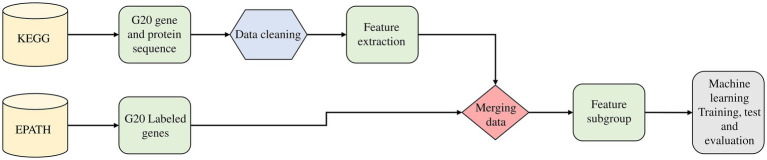
Emphasizing on the feature analysis step in the predictive model that involves the retrieval of annotated sequences of OA G20 from KEGG database, the feature extraction from the annotated sequences (gene/protein) and subsequently, feature subgrouping using EPATH.

Following our gene extraction from free text, we used our essential gene prediction model to check the status of each gene in our collection (Nembot et al., 2021). Figure 2 describes the predictive model and emphasizes the feature analysis step. In our model, identifying relevant features involved in each gene essentiality is of interest. This allows researchers to be able to generate new testable hypotheses.

### 2.3. Mapping and annotation of genes within *Oleidesulfovibrio alaskensis* G20

Primarily, UniProt database was explored to identify the proteins in genus *Desulfovibrio* that were encoded by the respective genes, and subsequently, NCBI GFF annotation file of OA G20 were used to locate the gene identifiers for the corresponding protein names. To provide functionality to the unannotated genes using identity-based approach, the nucleotide sequences of each unannotated genes were compared to nucleotide non-redundant databases using NCBI-BLASTP was employed (Altschul et al., 1997). The entire final set of genes were further characterized using NCBI-BLASTP, KEGG, and UniProt with their protein functionality, metabolic pathways, gene ontology, biological processes, and molecular functions.

### 2.4. Protein–protein interaction and enrichment analysis using STRING database and visualization by Cytoscape

The STRING (Search Tool for Interacting Genes Retrieval) database, which is a precomputation global resource for the prediction of functional association between the proteins was used to analyze the physical protein–protein interactions (PPIs; Szklarczyk et al., 2021). Eventually, it might be possible that the above set of genes may not be sufficient to address the objective of the study. Therefore, to enrich the essential gene list, the genes from individual pathways were analyzed using STRING database for protein–protein interaction (PPI) up to 2nd level. “*Desulfovibrio alaskensis* G20” was selected as the organism to enrich the gene set with the PPI enrichment value <1 × 10^−16^. The enriched gene set was then exported to Cytoscape with the help of StringApp (Cytoscape plugin; Shannon et al., 2003). The enriched gene set was clustered according to different pathways and visualized using the circular layout option. The STRINGdb text mining score and experimental score on the edges were noted to predict the strongness and weakness of the interactions between the nodes. In the PPI network, the nodes correspond to the proteins, and the edges represent the interactions.

### 2.5. Pangenome analysis

**Figure 3:**
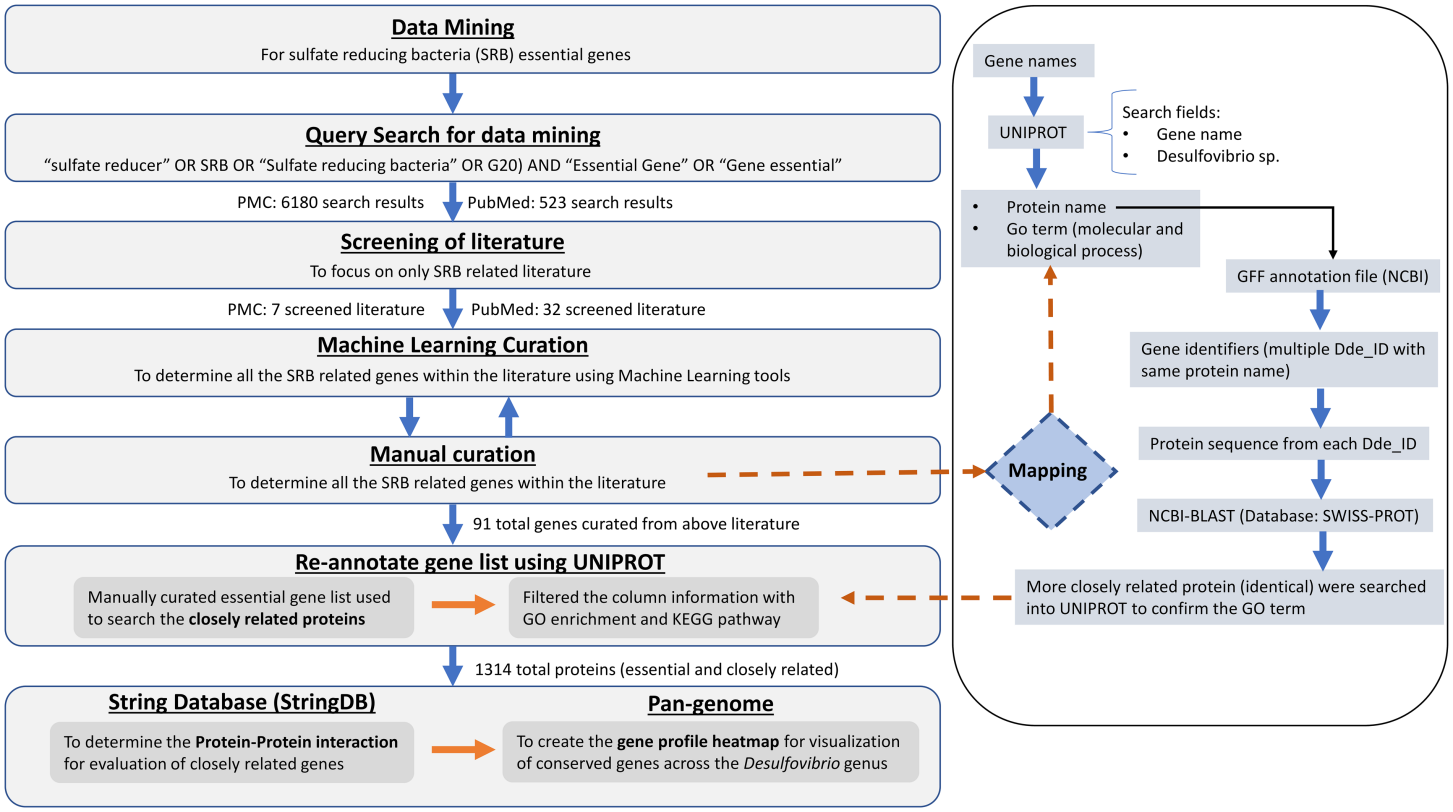
Overall workflow for the mining of essential genes of SRB from literature databases and subsequent identification of genes as essential in OA G20.

The text-mined gene sets were used to corroborate the results of the pangenome of 63 genomes of the genus *Desulfovibrio* developed by Shailabh et al. (under preparation). To develop a genus based pangenome, a total of 63 *Desulfovibrio* SRB genomes (.fna files) were downloaded from two different genome databases such as National Center for Biotechnology Information (NCBI) and JGI Integrated microbial genomes and microbiomes (JGI/IMG). These genome sequences were further processed for genome completeness and further annotated for uniform gene prediction and functional characterization. Subsequently, the FASTA files were uploaded on Google Colab (PPanGGOLiN), which resulted in the alignment of the genes with the SRB Pangenome. PPanGGOLiN pipeline has ability to build pangenomes for large sets of prokaryotic genomes through a graphical model and a statistical method to classify gene families into three classes: persistent, cloud, and one or several shell partitions. A set of annotated genomes with their coding regions classified in homologous gene families used a as input for analysis. PPanGGOLiN integrates information on protein-coding genes and their genomic neighborhood to build a graph where each node is a gene family, and each edge is a relation of genetic contiguity (two families are linked in the graph if they contain genes that are neighbors in the genomes). This model has an advantage of resilience to fragment genome assemblies for the pangenome structure. A gap in assembly of one genome can be supplemented by information from the other genome thus maintaining the link in the graph. The output file consisted of the corresponding gene families from pangenome, partition, and quality parameters in terms of percent identity (pident), e-value (expectation value) and bit score for each input gene. Consequently, the matching gene family matrix data was retrieved to visualize the presence/absence matrix (P/A) for the input gene list. Where rows correspond to gene families and the columns to genomes. Values are 1 for the presence of at least one member of the gene family and 0 for absence. This P/A matrix is modeled by a multivariate Bernoulli Mixture Model (BMM). Its parameters are estimated *via* an Expectation–Maximization (EM) algorithm considering the constraints imposed by the Markov Random Field (MRF). Each gene family is then associated to its closest partition according to the BMM. However, the interconnection of initial query search, manual curation of genes, re-annotation through gene and protein databases, PPI interactions of the gene sets, and the pan-genome across 63 *Desulfovirbio* genus is briefly described in the workflow as shown in Figure 3.

## 3. Result and discussion

### 3.1. Data mining and gene enrichment

**Figure 4:**
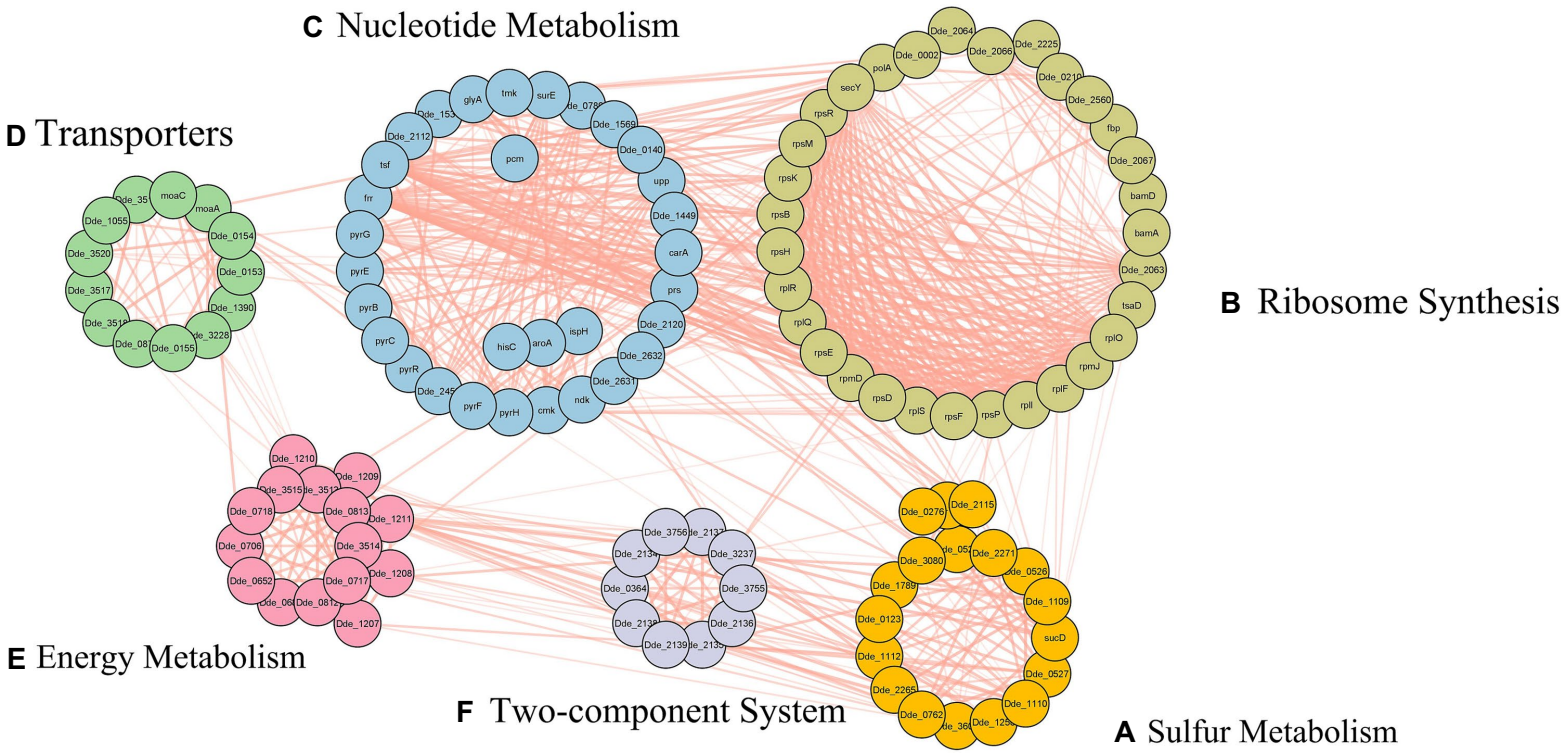
Protein–protein interactions of the enriched gene set, subcategorized under, **(A)** sulfur metabolism, **(B)** ribosome synthesis, **(C)** nucleotide metabolism, **(D)** transporters, **(E)** energy metabolism, and **(F)** two-component system.

A total of 39 articles (shown in Supplementary Table S1) were retrieved from both the databases (PubMed and PMC) and 91 genes (shown in Supplementary Table S2) were manually curated that belong to different *Desulfovibrio* genus. With OA G20 as the model organism, the total set of 91 genes were mapped and annotated on to the genome of the model organism and the chromosomal locus tag was noted. However, only 42 genes were observed to be present in the genome of OA G20 (shown in Supplementary Table S3), where 22 genes were either appeared twice or were of different name with same functionality (similar protein coding genes). The 22 genes were eliminated, and 20 genes were finally screened down for subsequent studies (shown in Table 1). The functions of these 20 genes were further validated through an extensive manual review of relevant literature, which involved various molecular strategies to confirm their essentiality (shown in Supplementary Table S3; Russel et al., 1990; Dolla et al., 2000; Goenka et al., 2005; Sarin and Sharma, 2006; Keller et al., 2009; Zane et al., 2010; Holkenbrink et al., 2011; da Silva et al., 2013; Krumholz et al., 2013; Theisen et al., 2013; Venceslau et al., 2014). The set of 20 genes were then sub-categorized into 6 major pathways namely – sulfur metabolism (8 genes), ribosome synthesis (1 gene), nucleotide metabolism (1 gene), transporters (3 genes), energy metabolism (5 genes), and two-component system (2 genes). The subset of 20 genes were further enriched to a total of 116 genes with the physical PPI interactions using String database (shown in Supplementary Table S4). The interactions and networks (shown in Supplementary Table S5) between the 116 enriched genes were visualized using Cytoscape and are shown in Figure 4.

**Table 1:** Annotated gene list subcategorized under different pathways.

Gene name	Gene ID	Gene ontology ID	Protein name
Sulfur metabolism [Dissimilatory sulfate reduction]			
*dsvB*	dde_0527	GO:0006790; GO:0009055; GO:0018551; GO:0020037; GO:0046872; GO:0051539	Sulfite reductase, dissimilatory-type beta subunit
*dsrA*	dde_0526	GO:0018551; GO:0020037; GO:0046872; GO:0051539	Sulfite reductase, dissimilatory-type alpha subunit
*cysL*	dde_3080	GO:0004124; GO:0006535	Cysteine synthase A
*aprA*	dde_1110	GO:0016491	Cysteine synthase A
*aprB*	dde_1109	GO:0046872; GO:0051536	Cysteine synthase A
*dsrC*	dde_0762	GO:0005737; GO:0018551	Sulfite reductase, dissimilatory-type gamma subunit
*CysH*	dde_1789	GO:0003824	Phosphoadenosine phosphosulfate reductase
*sat*	dde_2265	GO:0000103; GO:0004781; GO:0005524	Sulfate adenylyltransferase
Transporters			
*modB*	dde_3519	GO:0005886; GO:0015098; GO:0016021	Molybdate ABC transporter, inner membrane subunit
*modA*	dde_0155	GO:0015689; GO:0046872	Molybdenum ABC transporter, periplasmic molybdate-binding protein
*modC*	dde_3518	GO:0015689; GO:0030973; GO:0046872	Molybdenum ABC transporter, periplasmic molybdate-binding protein
Energy metabolism			
*cyc*	dde_0717	GO:0008863; GO:0008940; GO:0009055; GO:0042597; GO:0043546; GO:0045333; GO:0046872; GO:0047111; GO:0051539	Formate dehydrogenase, alpha subunit
*dsrM*	dde_0680	GO:0005886; GO:0009055; GO:0016021; GO:0022904	Cytochrome b/b6 domain-containing protein
*hmcB*	dde_0652	GO:0046872; GO:0051536	Formate dehydrogenase iron–sulfur subunit
*hdrA/qmoA*	dde_1209	GO:0016491; GO:0046872; GO:0051536	4Fe-4S ferredoxin iron–sulfur binding domain-containing
*hdrB/qmoB*	dde_1208	GO:0051912	Heterodisulfide reductase, C subunit
Ribosome synthesis pathway			
*trx*	dde_2066	GO:0004791; GO:0005737; GO:0019430	Thioredoxin reductase
Nucleotide metabolism			
*upp*	dde_1448	GO:0000287; GO:0004845; GO:0005525; GO:0006223; GO:0009116; GO:0044206	Uracil phosphoribosyltransferase
Two component system			
*HydA*	dde_2134	GO:0008901; GO:0009375; GO:0042597; GO:0046872; GO:0047806; GO:0051538; GO:0051539	Hydrogenase (NiFe) small subunit HydA
*HydB*	dde_2135	GO:0008901; GO:0016151; GO:0047806	Periplasmic (NiFeSe) hydrogenase, large subunit, selenocysteine-containing

**Figure 5:**
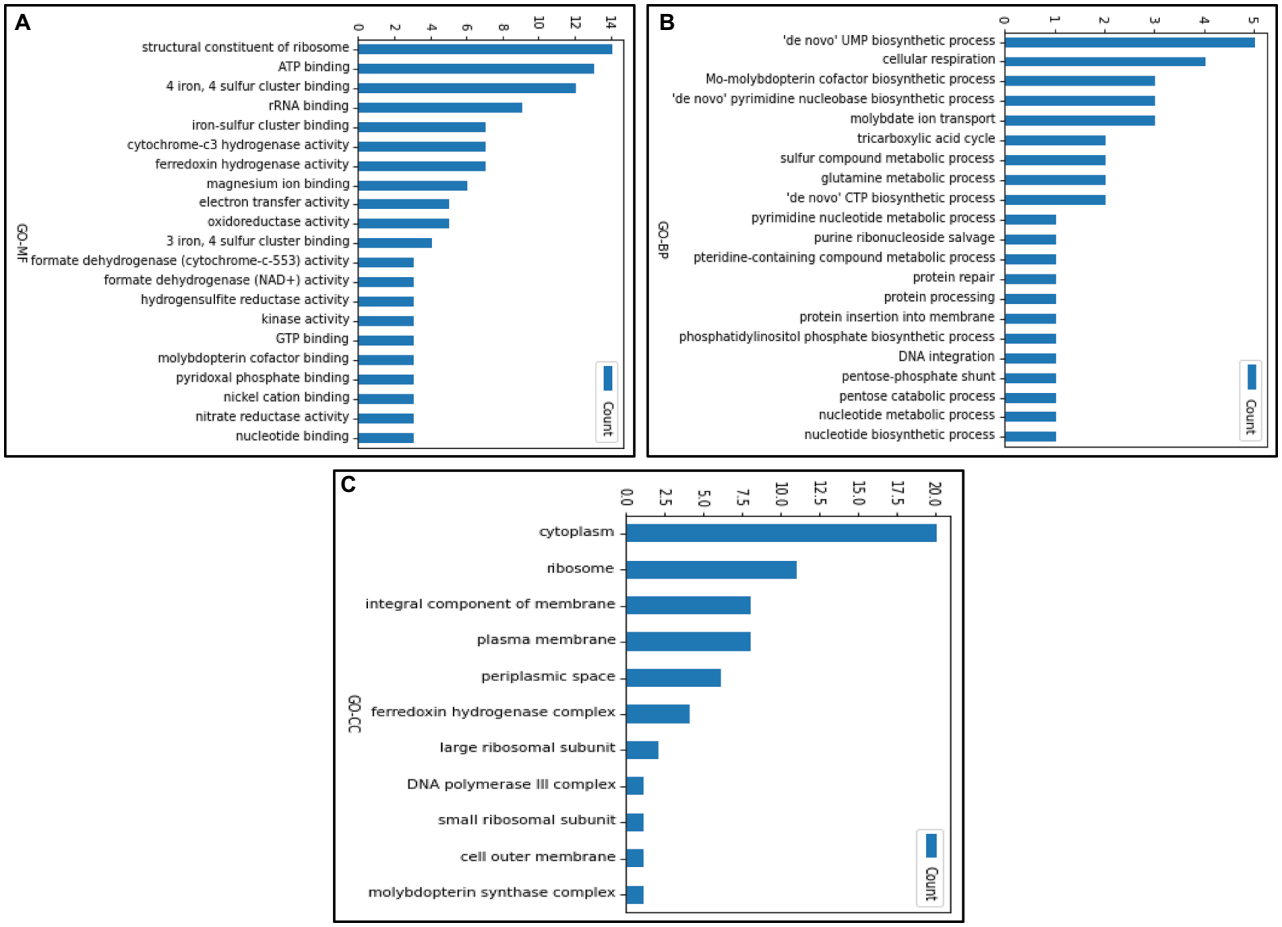
Gene count representing the individual GO terms in **(A)** molecular function **(B)** biological process **(C)** cellular component.

The gene ontology terms for each gene were retrieved from UniProt, and the frequency of genes corresponding to each GO terms is shown in Figure 5. The genes were categorized under three GO categories- molecular functions, biological processes, and cellular component. We observed that the highest gene count (14 genes) in molecular function falls under structural constituent of ribosome (ribosome synthesis). However, in the biological process, the highest gene count (5 genes) was observed in uridine monophosphate biosynthetic process (nucleotide metabolism). Furthermore, out of 116 enriched genes, only 20 genes were confined to the cytoplasmic region and 28 genes contributed to the membrane processes (facilitates influx and efflux transporting system through transporters). Using our text mining tool, we extracted 339 gene mentions with duplicates (gene mention in multiple locations) from our 39 papers. After duplicates removal and manual curation, 99 OA G20 genes were identified. Moreover, using our essential gene prediction model, we were able to predict that among the 99 genes, there were 19 non-essential genes (e.g., *dsrA*, *aprB*, *aprA*, *lapB*, *ccmC*, *cysH*, *metE*, *recA*, *cysK*, *cysE*, *cydA*, *cydB*, *cysQ*, *upp*, *metH*, *sat*, *metF*, *serA*, and *rpsN*) and 3 essential genes (*rpoA*, *rpoC*, and *rplF*). This signifies that the genes essential for the survival of OA G20 is environment dependent. Therefore, the remaining genes might be essential for different biologically relevant functions (as shown in our functional annotation below), which has been furthervalidated in our pangenome analysis. Our methodology relies on continuous learning and a text granularization algorithm as input to the model, which allows it to achieve better performance. Its evaluation process was done around BioCreative II annotated datasets (Smith et al., 2008). We obtained an average F1-score of 97.4%, which outperforms current methods like GeneNormPlus (Wei et al., 2015) and Hunflair (Weber et al., 2021; shown in Table 2).

**Table 2:** Comparative F1-score (Moyen and Meilleur) for machine learning based models.

	Tools	Moyen score-F1	Meilleur score-F1	References
1	GeneNormPlus	68.6	87.1	Wei et al. (2015)
2	HunFlair	80.61	87.71	Weber et al. (2021)
3	Our method	89.36	97.40	Fotseu et al. (2021)

### 3.2. Sulfate metabolism pathway

SRBs are prevalent in both natural and concocted environment and makes use of the oxidized form of sulfur as an electron acceptor, preferably sulfate, to obtain energy from organic compounds such as lactate and formate for their anaerobic survival (Qian et al., 2019). The fate of the sulfate reduction is strictly governed by a series enzymatic machinery; the genes of which are present within the dissimilatory sulfate reduction pathway. The first step of dissimilation initiates with the reduction of sulphate to sulfite, and subsequently to sulfide (Pott and Dahl, 1998). The free sulfate group attaches to two molecules of adenosine triphosphate by replacing one phosphate to form adenosine phophosulfate (APS; Ullrich et al., 2001). The former reaction involves the activation of free sulfate using APS sulfurylase, which is encoded by the sulfate adenylyltransferase (*sat*) gene in SRBs (Kushkevych et al., 2020). Thereafter, in the ensuing step, the activated sulfate is being reduced by APS reductase by the consumption of two electrons and generation of adenosine monophosphate (AMP; Barton et al., 2014). With more interest that has been paid through years to elucidate the mechanism behind the reduction of sulfite to sulfide, Fliz and Cypionka proposed an alternative hypothesis which involves a sequel of enzymes mainly – sulfite reductase, trithionate reductase, and thiosulfate reductase (Cypionka, 1995). In several *Desulfovibrio* genus, the *in-vitro* assays have confirmed the involvement of dissimilatory sulfate reductase (*dsrA* and *dsrB*) for the reduction of sulfite (Santos et al., 2015). At first, *dsrAB* participates in the reduction of sulfite to *dsrC* trisulfide; afterwards the reduction of the same is being triggered by *DsrMKJOP* complex (Ferreira et al., 2022). The sulfite to sulfide reduction is the last step in the sulfate reduction pathway, where the reactive sulfite is released to the environment as toxic sulfide (Kemp and Thode, 1968). As reported in few experiments, being the physiological partner of *dsrAB*, the absence of *dsrC* resulted into partial reduction of sulfate and the dominancy of thiosulfates were detected (Leavitt et al., 2019). Therefore, the dissimilatory reduction of sulfate *via* the reduction of sulfite to sulfide, *dsrAB* along with *dsrC* were reported to be the central and potentially essential proteins during sulfate metabolism (Anantharaman et al., 2018). In addition, adenylylsulfate reductase is a heterodimeric complex of two subunits, *aprB* and *aprA*, which is conserved among the sulfate reducers and is considered as a key enzyme in the dissimilatory sulfate reduction (Meyer and Kuever, 2007). *dsrD* enhances the activity of *dsrBA* and is often used as a functional marker to assign the type of sulfur energy metabolism in sulfate reducers (Hittel, 1998).

In our study, using the query search we retrieved 8 genes from sulfate reducing pathway- namely, *dsrB*, *dsrA*, *dsrC*, *cysl*, *aprA*, *aprB*, *sat*, and *cysH*. These genes were further enriched with the PPI enrichment *p*-value of 3.33 × 10^−16^ and an interaction network containing a total of 18 genes was observed (shown in Figure 4A). The enriched genes (dde_0276, dde_0528, dde_1112, dde_1258, dde_2115, dde_2271, dde_3081, dde_3604, *cysQ*, and *sucD*) were not only unconstrained to sulfate metabolism but a strong network to three other pathways- cysteine and methionine metabolism, selenocompound metabolism, and secondary metabolite synthesis pathway were observed. The *sat* gene (dde_2265) was observed to be the bridging gene between all the enriched pathways. To claim the above observation with regard to the mechanism behind the association of *sat* gene with all the four pathways there is a need to understand the assimilatory sulfate reduction (ASR) pathway. In ASR pathway, the crucial metabolites at the end step of the intermediate reactions are the sulfur-containing amino acid- cysteine and methionine (Courtney-Martin and Pencharz, 2016). In one of the intermediate steps, cysteine serves as the sulfur donor for methionine (Mueller, 2006). On a broader aspect, amino acid metabolism pathways are essential for the survival of organisms because it coined itself as the building block for proteins and enzymes. In OA G20, from the interaction network, we observed that cystathionine beta-lyase (dde_0726) was enriched, which catalyzes the production of homocysteine, a direct precursor metabolite for the formation of methionine (Gamrasni et al., 2020). Parallelly, methionine synthase (dde_2115) was also enriched, which is a vital enzyme in the intermediate step of methionine synthesis (Ekstrom et al., 2003). Serine O-acetyltransferase (dde_3081) shares its role with the Seleno-compound metabolism along with the cysteine metabolism, which itself derives from serine (Turanov et al., 2011).

With the involvement of more complex enzymatic machinery the ASR pathway comprises of *cysQPUWA* operon that encodes for sulfate permease, *cysDNC* that encodes for ATP sulfurylase, *cysH* gene for phosphoadenine phosphosulfate reductase (from the operon *cysJIH*), and *cysK* and *cysM* genes for O-acetylserine-(thiol)-lyase. The *cysI* and *cysH* genes of the ASR pathway were retrieved initially and the enrichment of cysQ was observed (Abola et al., 1999). The quinone-interacting membrane-bound oxidoreductase that is encoded by the gene dde_1112 is responsible for the electron transport to maintain electron flux within sulfate reducers (Keller et al., 2014). Keller et al., showed that deletion of this gene resulted in the inhibition of growth in *Desulfovibrio vulgaris*, where an interesting fact was observed signifying its importance for the function of Coo hydrogenases in the transport system, which is reported to be crucial for the cellular growth in lactate and sulfate (Calisto et al., 2021). Fumarate reductase cytochrome b subunit (dde_1258) participates in the formate respiration using sulfide as the electron donor and depicted the role as the extracellular electron transfer within the two-component system along with the electron transport chain in sulfate reducers (Reguera, 2018). Moreover, D-lactate dehydrogenase (dde_3081) is responsible for the regular elector donating mechanism from lactate by releasing two electrons, and indirectly through the assimilation and dissimilation produces ATP and sulfide, respectively (Pohanka, 2020).

Succinyl-CoA synthetase (*sucD*) was enriched which is usually a part of the citric acid cycle that produce succinates (succinic acid) and are considered as a secondary metabolite in the anabolic process during respiration (Li et al., 2013). Subsequently, in addition to the same pathway, the production of folic acid as a secondary metabolite is also crucial as it is required for the protein and nucleic acid synthesis involved in the cell division and growth of the organism (Fernandez-Villa et al., 2019). The secondary metabolites are crucial for the cell survival to withstand harsh environments and therefore, the genes associated with this pathway are conditionally essential for the existence of the organism (Moses et al., 2017).

### 3.3. Ribosome metabolism pathway

From the aspect of central dogma, the codons, which are a group of three nucleotides, are converted to amino acid and subsequently to proteins using an intricate ribonucleoprotein machine, the ribosome (Popel et al., 2020). In cells, ribosome acts as the workbench for protein synthesis by the process known as translation (Gallie, 2002). Proteins are the integral elements of a cell that contribute wholly towards the cell survival by determining the structure, regulation, and reaction metabolism within the cell (Jacob and Monod, 1961). In the cytoplasm, ribosomes consist of the protein synthesis machinery, which fulfills its activity through four main phases: initiation, elongation, termination, and recycling (Kaczanowska and Rydén-Aulin, 2007). Therefore, the foremost step is the synthesis of ribosome, termed as ribosome biogenesis, which takes place from the transcription of several genes from ribosome operon (Singh et al., 2016). In prokaryotes, especially in bacteria, the ribosome (70S) is composed of two disproportionate subunits- the 30S and the 50S subunit, which gather at the ribosome binding site on the mRNA during translation initiation, where each subunit contributes to specific functions in protein synthesis (Moll et al., 2004). The 30S subunit is comprised of 21 ribosomal proteins along with 16S rRNA and is responsible for decoding mRNA. However, the 50S subunit is arranged with 33 ribosomal proteins, the 23S rRNA, and the 5S rRNA, and operates to assist tRNA–amino acid monomers, catalyze peptide bond formation, and excrete polypeptides. The *rpl* and *rpm* operon codes for 50S ribosomal subunit, whereas *rps* operon codes for 30S ribosomal subunit. There also exists additional accessory operon that facilitate the functioning of the ribosomal subunits. Moreover, the genes associated with the synthesis pathway are crucial for clustering and folding of the proteins (Javed et al., 2017).

Around 55 ribosomal proteins are conserved among the bacterial species, and the number is fairly constant. In our study, with the initial query of the gene dde_2066, there were enriched genes which fell under the ribosomal operon, namely, *rpsBDEFHKMPR*, *rplFIOQRS*, *rpmDJ*, *bamAD*, *fbp*, and other genes including *secY*, *tsaD*, dde_0002, dde_0210, dde_2063, dde_2064, dde_2066, dde_2560, dde_2225 and dde_2067 (shown in Figure 4B). Besides, the stronger interaction within the ribosomal gene set, an immense association between these genes and the gene set from the nucleotide metabolism pathway were observed. Several reports from bacterial genome-wide annotations exhibited the absence of some ribosomal proteins in tiny genomes and the unaltered cellular phenotype is suggestive of the fact that a few ribosomal proteins may be non-essential for the organisms’ survival (Hunt et al., 2006). The genes from the operon *rplM-rpsI*, *rplU-rpmA*, and *rpmB-rpmG* that encodes for L13–S9, L21–L27, and L28–L33, respectively are significantly essential for ribosome assembly and functioning (Aseev et al., 2016). Literature showed that L27-deficient strain may show adverse effects on growth due to non-functional 50S subunit (Wall, 2015). L13 has been reported to be the utmost essential protein which interacts with 23S RNA at an early stage *via srmA*, which is also an essential foundation protein towards the assembly of 23S RNA. The 50S ribosomal gene *rpmJ*, which encode for L36 is quite essential for the expression of *secY* gene (encodes an essential transmembrane protein known for translocation activity at the periphery of ATPase motor domain) and is located at the downstream region of the *spc* operon (encode 11 ribosomal proteins along with *secY*; Ikegami et al., 2005). Nevertheless, Ikegami et al., showed that knockout of *rpmJ* gene in *E. coli* resulted in altered expression of *secY* gene and observed inhibition in protein translocation. The tsaD in cooperation with TsaB and TsaE, transfers the L-threonylcarbamoyl-moiety from threonylcarbamoyladenylate onto tRNA and is essentially responsible for translation fidelity (Ikegami et al., 2005). Therefore, the enriched operon and its genes are of utmost essential for the regulation of translation processes (clustering and folding of proteins, and coding mRNA to form codons and decoding signals for t-RNA amino acid attachment) in central dogma.

### 3.4. Nucleotide metabolism

Nucleotide metabolism is vital in maintaining the bacterial physiology and producing the nucleic acids necessary for DNA replication and RNA transcription (Lopatkin and Yang, 2021). Moreover, nucleotide metabolism is directly linked to cellular homeostasis as it is essential for physiological processes such as carbohydrate metabolism, oxidative phosphorylation, essential nucleotide biosynthesis, and signal transduction (Gomes and Blenis, 2015). The nucleotide metabolism pathway leads to the synthesis of purines and pyrimidines, which are the major energy carriers, subunits of nucleic acids (DNA and RNA) and precursors for the synthesis of nucleotide cofactors such as NAD and SAM (Kilstrup et al., 2005). The production and regulation of these biological macromolecules are essential for survival and replication of organisms (Woolf, 2015). Furthermore, nucleotide synthesis requires the vitamin folic acid, the enzyme for which was observed to be enriched from the sulfate energy.

In our study, the PPI network of the genes from the nucleotide metabolism showed higher enrichment with the initial of 1 gene (upp) to 31 genes (*p*-value: <1.0 × 10^−16^). The enriched genes fall under *pyrBCEFGHR*, *tmk*, *tsf*, *prs*, *pcm*, *ndk*, *frr*, *cmk* operon and other genes which include *surE*, *aroA*, *carA*, *glyA*, *hisC*, *ispH*, dde_0789, dde_0140, dde_1449, dde_1537, dde_1569, dde_2112, dde_2120, dde_2453, dde_2632, dde_2631 (shown in Figure 4C). In this metabolism, most of the genes were observed in the purine and pyrimidine metabolism, biosynthesis of amino acid, biosynthesis of secondary metabolites, and nicotinate and nicotinamide metabolism pathway. The *pyr* operon consists of genes that encode proteins necessary for pyrimidine biosynthesis. In general, *pyrR* regulon mediates the regulation of the *pyr* operon using the attenuated termination or anti-termination processes *via* RNA switch. The expression performance of pyrR is maintained by the presence of a certain concentration of uracil, which gets produced by *upp* gene (encodes for uracil phosphoribosyltransferase; Romby and Charpentier, 2010). The *pyrB* and *pyrI* are the contiguous genes that encode for the six catalytic and six regulatory chains of aspartate carbamoyltransferase, respectively, and are transcribed from the same promoter. The operon pyrBI maintains the reiterative transcription and the deletion-mutation of the promotor of this operon or the pyrBI abolishes the synthesis of pyrimidine (Pauza et al., 1982). The *pyrP* that encodes uracil permease is a transporter that facilitates the entry and exit of nucleosides or uracil during the biosynthesis process (Sanchez and Demain, 2008). The *pyrH*, which codes for uridylate kinase is reported to be phenotypically essential when deletion-mutation was performed by Koo et al. (2017). Interestingly, the mutation of *pyrF* gene, which encodes for orotidine 5-phosphate decarboxylase, results in poor growth of the organism (Oakley et al., 1987). However, the *pyr* operon is not limited to the pyrimidine synthesis or salvage but has widespread role in the synthesis of essential amino acids such as glutamine, histidine (*prs* and *HisC* histidine biosynthesis) and arginine, to name a few. This genes from pyrimidine synthesis genes are directly interconnected to *cmk* (codes for the synthesis of cytosine triphosphate), and tmk (codes for biosynthesis of thymine triphosphate), along with the *ndk* gene which, maintains the equilibrium concentration between different nucleoside triphosphates (Moat and Foster, 2002). The interlinked functionality and co-expression of the above genes leads to generation of nucleotides to synthesize deoxyribonucleic acid (DNA) and ultimately preserves the normal cellular growth and is therefore suggestive of the fact that each individual gene from the nucleotide metabolism is crucial for the normal growth pattern of the organism.

### 3.5. Transporters

The cellular library of transporter proteins is accountable for both the uptake of essential nutrients such as carbohydrates, amino acids, and metals into the cell, as well as the effluence of toxins and antimicrobial agents out of the cell (Bolhuis et al., 1997). Moreover, to maintain an equilibrium condition in any living organism, there is a need of transporter machinery to facilitate organic and inorganic molecules across cellular membranes (Wilkens, 2015). The ABC transporters are known to be crucial hence they share a common function and a common ATP-binding domain to make up a large superfamily of proteins (Rees et al., 2009). Michael et.al reported in their study that gram negative bacteria majorly comprise of two major export system – (1) the ABC transporters, and (2) pullulanase-like family of transporters. In our study we enrich a list of 13 genes (dde_0153, dde_0154, dde_0155, dde_0871, dde_1055, dde_1390, dde_3,228, dde_3517, dde_3518, dde_3519, dde_3520, *moaA,* and *moaC*). These enriched genes (shown in Figure 4D) majorly comprise of molybdate transport proteins. These transporter proteins majorly regulate the transport of molybdate by an ABC-type transporter, comprise of three proteins- *modA*- periplasmic binding protein, *modB*- membrane protein and *modC*- the ATPase (Locher, 2009). Since molybdate is an essential trace element required in the growth medium of SRB, there is a need to transport molybdenum inside the cell (Smedley and Kinniburgh, 2017). Molybdoenzymes such as nitrate reductases and dimethyl sulfoxide reductases are present in elevated concentrations during the anaerobic growth which makes molybdenum cofactor crucial for anaerobiosis (Anderson et al., 2000).

### 3.6. Carbon and energy metabolism

The energy and carbon metabolism consists of particular central pathways which are highly preserved across the variety of bacterial species, regardless of the wide variety of growth substrates and conditions they utilize (Sudarsan et al., 2014). Certain conserved central reactions are almost universally present in many species for provision of carbon frameworks for biosynthesis (Satanowski et al., 2020). Bacterial energy requirements are met by substrate level phosphorylation reactions, along with the membrane bound ATPase and the transmembrane proton gradient (Kakinuma, 1998). In addition to carbon requirements, bacteria have nutritional requirements for nitrogen, oxygen, phosphorous, sulfur and a variety of other elements necessary for cellular functions (Merchant and Helmann, 2012). However, for the sulfate reduction in SRB of the genus *Desulfovibrio*, generally use organic acids such as lactate, pyruvate, and hydrogen as electron donors.

In our study with the initial gene set of 5 genes *cyc, dsrM, hmcB, hdrA/qmoA, hdrB/qmoB*, we enriched a total of 15 genes and other 10 genes include- dde_0706, dde_0718, dde_0812, dde_0813, dde_1207, dde_1210, dde_1211, dde_3513, dde_3514, dde_3515 (shown in Figure 4E). As discussed in sulfur metabolism pathway, we know APS reductase is crucial in the conversion of sulfite to sulfide by the process of dissimilatory reduction pathway. Several literatures reported that the *qmoABC* complex often resides with *aprAB* genes. Duarte et al. showed the direct electron transfer between the OA G20 *qmoABC* complex and *aprAB* indicating that in the absence of *qmoABC* complex, APS reduction was not observed (Duarte et al., 2016). Interestingly, Zane et al., showed the linkage of *qmoABC* operon with sulfate reduction genes (*apsBA*) and the involvement of *Qmo* proteins in electron delivery to APS reductase. The mutant of *Desulfovibrio vulgaris* lacking *qmoABC* operon was not capable to grow on medium containing sulfate as a sole source of electron acceptor along with reductant source such as lactate, formate, pyruvate, ethanol, or hydrogen (Zane et al., 2010). This reports the essentiality of *qmoABC* operon in the survival of sulfate reducing bacteria by way of sulfate respiration. Another operon which includes in this dataset involves the high molecular cytochrome (hmc) complex which is involved in the electron transport linkage of periplasmic hydrogen oxidation to cytoplasmic sulfate reduction (Pereira et al., 2007). The hmc complex is a nine-heme cytochrome complex and is inevitably combined with *dsrMJKOP* operon, where *dsrM* is localized at the transmembrane region and consist of FeS, b- and c- type cytochromes, which functions to regenerate the *dsrC*-sulfur carrier protein (Grein et al., 2010). The *cyc* gene which encodes the cytochrome-c3 is an integral part of *dsr* operon and belongs to the *hmc* family protein. Keon et al. showed the involvement of *hmc* operon in electron flow from hydrogen to sulfate (Keon et al., 1997). Similarly, Dolla et al. (2000) showed that the *hmc* operon is not only involved in electron transport from hydrogen to sulfate, but also essential for the low-redox potential niche that helps the single cells to form colonies where hydrogen is solely present as the electron donor for sulfate reduction (Dolla et al., 2000). Therefore, *hmc* operon is crucial for hydrogen dependent growth in SRB.

### 3.7. Two component system

Two component regulatory system plays vital role in sensing and responding towards internal and external environment factors and are the most important signaling systems, which helps the bacterial cells to control essential and secondary physiological processes (Mitrophanov and Groisman, 2008). However, in the fluctuating environment condition or in stress conditions, bacterial cells often require stimulus perception and the successive modulation of the expression of relevant genes to optimize metabolism and physiology for their survival (Papadimitriou et al., 2016). In the process of hydrogen cycling, hydrogen plays central role as an intermediate in the generation of a chemiosmotic gradient from the oxidation of organic molecules (Kulkarni et al., 2009). The cytoplasmic [NiFe] hydrogenases along with the [FeFe] hydrogenases are widely dispersed among the SRBs, and a few other hydrogenases such as formate dehydrogenases, and heterodisulfide reductase-related proteins are potential candidates that partakes in the energy coupling mechanism using electron splitting, for which H_2_, formate, pyruvate, and NAD(P)H, β-oxidation serves as an electron donor (Finney and Sargent, 2019). Caffery et al., performed mutation-deletion on each individual hydrogenases, where the expression data of *hyd, hyn1, hys,* or *hyn1* and *hyd* mutants when grown under the condition of high and low lactate/H_2_ concentrations suggested that [NiFeSe] hydrogenase is critical for growth at lower concentrations of hydrogen, while the [Fe] hydrogenase eases enhanced growth at higher hydrogen and lactate concentrations (Pohorelic Brant et al., 2002). Most of the genes that belong to periplasmic hydrogenases help in the oxidation of molecular hydrogen. These genes include – dde_2134, dde_2135, dde_2137, and dde_2138 which encodes for [Ni-Fe]-hydrogenases present in twin-arginine translocation pathway, periplasmic (NiFeSe) hydrogenase containing large subunit, periplasmic (NiFe) hydrogenase containing small subunit and [NiFe]/[NiFeSe] hydrogenase large subunit family, respectively (Valente et al., 2006).

In SRB, hydrogen is known to be keenly involved in sulfate reduction pathway as at the substrate level phosphorylation, under the influence of organic substrates, H_2_ from either outside the cell or from the cytoplasm can act as the direct electron donor to dissimilatory sulfate reductases – *dsrA* and *dsrB* (Karnachuk et al., 2021). The regularity has been observed in our study, where we found that among the 12 genes enriched in two component system (shown in Figure 4F), the two genes dde_0526 (*dsrA*) and dde_0527 (*dsrB*) shared strong interactions with the two-component system. Therefore, with regard to the foreplay between the hydrogenases and sulfate reductases, the cytoplasmic and periplasmic hydrogenases mediated extracellular electron transfer plays a crucial role towards ATP generation, which is critical for the survival of the organism, and hence hydrogenases may potentially be considered as essential (Cook et al., 2014). Nevertheless, two component system of SRBs comprises of several histidine kinases and response regulators, the majority of which belong to the periplasmic region, senses external environment using the transmembrane domains, and transduces signal to the cytoplasmic enzyme domains to subsidize survival, habituation and adaptation to the environment (Fabret et al., 1999). However, no interactions were observed between the kinases or regulators, and the genes of periplasmic hydrogenases.

### 3.8. Pangenome analysis to determine the conservation of genes across different *Desulfovibrio* genomes

**Figure 6:**
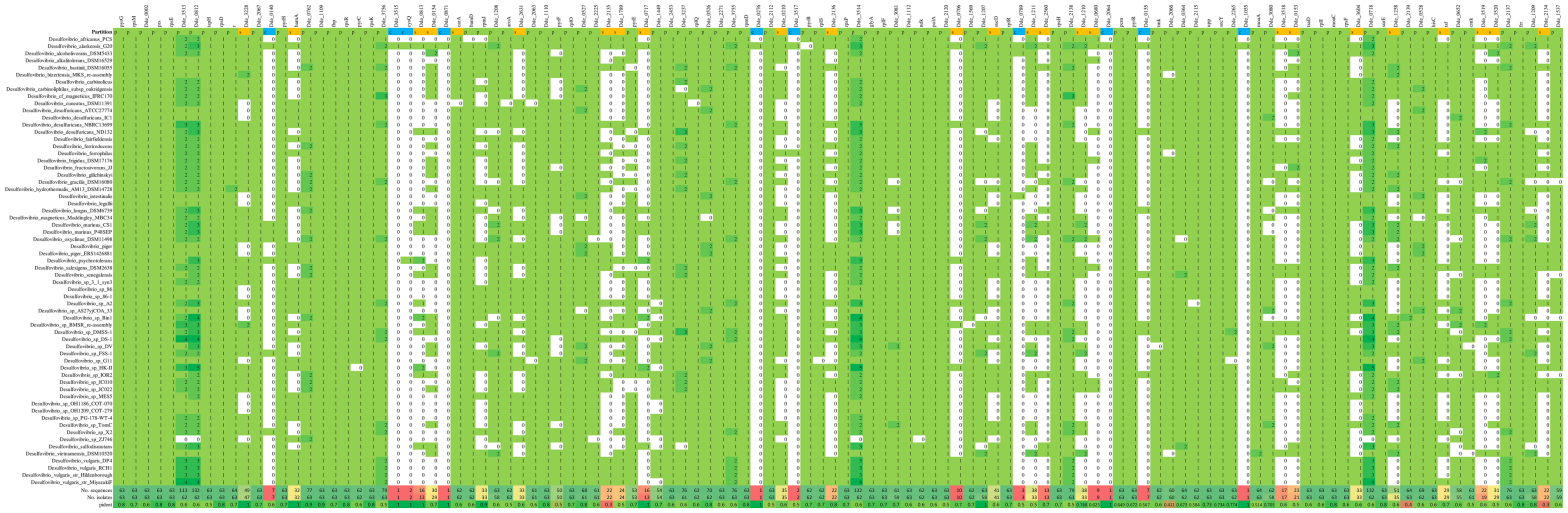
Heatmap showing the presence-absence matrix for all the conserved genes across the 63 different *Desulfovibrio* genomes. The vertical columns denote the enriched genes, and the horizontal rows denotes the *Desulfovibrio* genomes. The green and the white background denotes the presence and absence of the enriched genes in corresponding genome, respectively.

The analysis using pan-genome confines the genes which are conserved throughout a group of genomes from specific genus and can certainly be subcategorized under persistent, shell and cloud. In general, the genes that belong to the persistent category are highly conserved and are being shared across the genomes with very low or negligible mutation rates; the genes from this category are commonly known as orthologous genes (Rouli et al., 2015). Therefore, the “persistent” genes may be considered as the most crucial in some or the other survival mechanisms in the organism. The genes which are not as much conserved as compared to the genes from the persistent category, can be grouped under the category of “shell.” The genes from the shell category can be a part of several evolutionary advancement, and the evolutionary deletion-mutation of these genes in some genomes facilitates the organism to survive under changeable environment (Snipen and Ussery, 2010). The “cloud” or the peripheral genes are the minimal set of genes that may be present in either one or a few genomes and are not considered as essential because of their inextricable relation with the ecological alteration (Beier and Thomson, 2022). In our study, we validated the 116 enriched gene set by verifying their conservation across the 63 *Desulfovibrio* genomes using pangenome as a tool. The presence-absence matrix (heatmap) of the enriched genes across the *Desulfovibrio* genomes are shown in Figure 6 and Supplementary Table S6.

**Figure 7:**
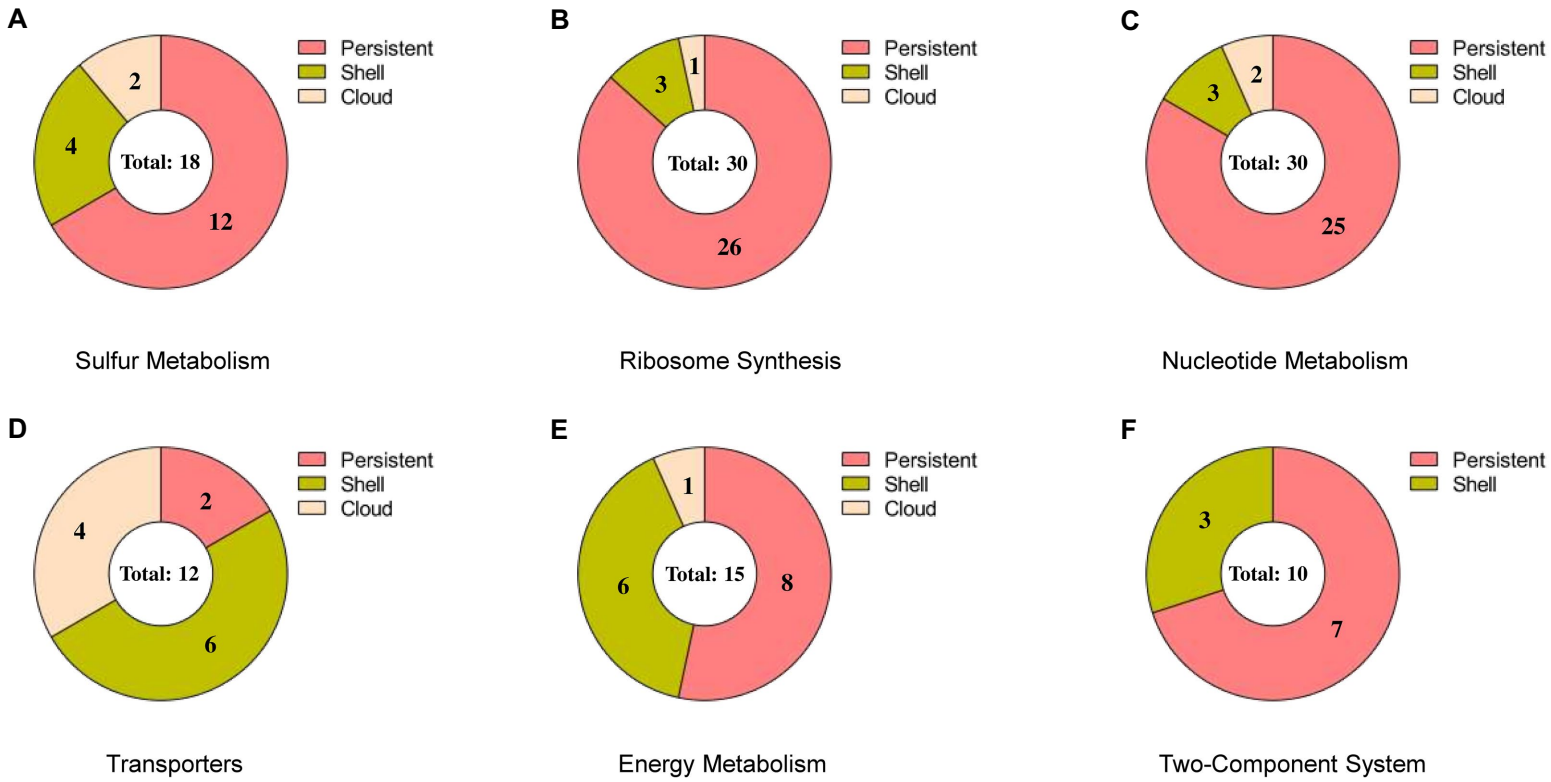
Pie chart representing the subgroups of a pan-genome (persistent, shell, and cloud). Each chart represents the sub-categorized genes in **(A)** sulfur metabolism, **(B)** ribosome synthesis, **(C)** nucleotide metabolism, **(D)** transporters, **(E)** energy metabolism, and **(F)** two-component system.

The pathway categorized enriched genes were further sub-grouped under persistent, shell and cloud with reference to the presence-absence matrix and are shown in Figure 7. We observed that the maximum number of genes (81 genes, 69.83% of the total) in any pathway except for transporters, comes under persistent category. However, a significant number of genes (25 genes, accounting for 21.55% of the total) were observed to be present in the shell and a very few in cloud category (10 genes, accounting 8.62% of the total). The absence of the genes from shell and cloud category in genomes may concede their tangled relationship with the ecological adaptation, which signifies that these genes are only “essential” under particular ecological alteration. The genes from the persistent, shell and cloud categorized into respective pathways are listed in Table 3.

**Table 3:** Segregation of the genes based on the pangenome analysis (persistent, shell and cloud).

Pathways	Persistent (P)	Shell (S)	Cloud (C)	Number of genes			
				P	S	C	Total
Sulfur metabolism	dde_0762, dde_1109, dde_1110, dde_0527, dde_0526, dde_2271, dde_3081, dde_1112, dde_2115, dde_2265, dde_3080, and dde_0528	dde_1789, *sucD*, dde_3604, and, dde_1258	dde_0123, dde_0276	12	4	2	18
Ribosome synthesis	*rpsM, rpsE, rpsD, fbp, rpsR, rpsK, bamD, rpmJ, rplO, rplQ, rpmD, rplS, rpsP, rplF, polA, rplR, rpsH, secY, tsaD, rplI, rpsF*, dde_0002, dde_2067, dde_2063, dde_2225, dde_1390, and dde_2066	*bamA*, dde_0210, and dde_2560	dde_2064	27	3	1	31
Nucleotide metabolism	*pyrG, prs, ispH, pyrH, pyrC, aroA, pyrF, pyre, pyrB, glyA, ndk, frr, pcm, pyrR, tmk, upp, surE, hisC, cmk*, dde_1449, dde_2453, dde_2112, dde_1537, dde_2120, and dde_1569	*carA*, dde_2631, *tsf*	dde_0140, and dde_0789	25	3	2	30
Transporters	*moaA,* and *moaC*	dde_3,228, dde_0154, dde_3518, dde_0513, dde_3519, and dde_3520	dde_0871, dde_3517, dde_0155, and dde_1055	2	6	4	12
Energy metabolism	dde_3513, dde_0812, dde_1208, dde_3514, dde_1207, dde_0718, dde_0652, and dde_1209	dde_0813, dde_0717, dde_0706, dde_1211, dde_1210, and dde_0680	dde_3515	8	6	1	15
Two-component system	dde_3756, dde_3237, dde_3755, dde_2138, dde_0364, and dde_2139, dde_2137	dde_2135, dde_2136, and dde_2134	NA	7	3	0	10
				81	25	10	116

In sulfur metabolism, we observed that out of 18 genes, the 12 genes that are present in the persistent are of utmost importance towards the survival of the organism (shown in Figure 7A). As observed from the biochemical mechanisms of sulfate reduction, the *dsr* operon was of utmost importance for the survival of OA G20 in presence of sulfate as electron acceptor. Along with the *dsrABC* genes, we observed that the *dsrABC* genes are persistent and are present in all the *Desulfovibrio* genomes. However, dde_1789, which is present in shell and codes for phosphoadenosine phosphosulfate reductase (catalyzes the formation of sulfite from adenosine 5′- phosphosulfate) is absence in 39 genomes signifying that this gene is non-essential when APS reductase (dde_1109, in persistent) is present in the genome (carries out the same function). The presence of the genes *sucD* (citric acid cycle), dde_3604 (lactate dehydrogenase, catalyzes the lactate to pyruvate conversion), and dde_1258 (fumarate reductase; citric acid cycle) in the shell (absent in 22, 29, and 29 genomes, respectively) signifies that these genes are only essential when the organism is grown under lactate as the carbon source. In conclusion, with lactate as an electron donor and sulfate as an electron acceptor for OA G20, the genes from the persistent, shell and cloud except dde_1789 is essential for its survival.

In ribosome synthesis, only 3 genes (*bamA*, dde_0210, and dde_2560) and 1 gene (dde_2064) were present in shell and cloud, respectively (shown in Figure 7B). *bamA* is an essential protein because it assembles the β-barrel in the outer membrane and is reported to be conserved across the gram-negative bacteria. The absence of *bamA* in some *Desulfovibrio* genomes may be due to its poor annotation in those genomes. dde_0210, and dde_2560 that encodes for thioredoxin disulfide reductase, and peroxiredoxin is only essential when the organism is under oxidative stress. The functionality of dde_2064 is unknown and thus the absence of this gene is difficult to conclude.

In nucleotide metabolism (shown in Figure 7C) the genes- *carA*, dde_2631, *tsf*, dde_0140, and dde_0789 are present in shell and cloud; the deletion of these genes may not stop the organism’s growth but will hinder the mobility and results in cellular phenotypic alterations. Butcher et al., showed that the deletion of *carA* in *Pseudomonas syringae* resulted in arginine and pyrimidine auxotrophy. In transporters (shown in Figure 7D), only *moaA* and *moaC* (molybdopterin biosynthesis) are present in persistent category, and most of the genes (molybdenum permeases, and transferases) are in shell category (Butcher et al., 2016). This suggests that molybdenum as cofactor is essential for OA G20, and other *Desulfovibrio* species but not for all *Desulfovibrio* species.

In energy metabolism (shown in Figure 7E), dde_0813, and dde_0717 encode for formate dehydrogenase, and dde_0706 encodes for formate dehydrogenase accessory protein and are present in shell category; both are only essential when formate is used by the organism as the electron donor. dde_1211 (a ferredoxin binding protein of unknown functionality), dde_1210 (a hydrogenase delta subunit), and dde_0680 (a cytochrome b-b6 domain containing protein and involves in electron transport chain), are not well established to conclude their presence-absence in anaerobic genomes.

Interestingly, no genes were found in cloud category in two-component system (shown in Figure 7F) because this complex system is essential for signaling transduction in cells by sensing the environment, but the three genes, dde_2135 (periplasmic NiFeSe hydrogenase), dde_2136 (hydrogenase maturation protease), and dde_2134 (cytochrome c3 hydrogenase) are present in shell. The genes that are present in the shell are not essential if hydrogen is not the electron donor, or the organism is not growing in presence of hydrogen.

Using manual curation and predictive model, we identified gene sets that are crucial for our model SRB, OA G20, and validated these genes with the results of pangenome analysis. Our results showed that the essentiality of the genes was environmental dependent. *Per se*, the sulfate reduction pathway is of utmost important for the respiration of OA G20 in environments where sulfate is the sole electron acceptor, and all the genes present in dissimilatory and assimilatory pathways were observed to be essential. The ribosomal genes are essential for the formation of large and small ribosomal subunits, but in OA G20, the presence of thioredoxin disulfide reductase and peroxiredoxin is only crucial to withstand oxidative stress. Molybdenum is an essential cofactor for the growth of OA G20, and as observed, its transporter proteins are highly essential. Lactate is a well-known electron donor for SRB (and OA G20), and therefore, the presence of lactate dehydrogenase in essential gene list is obvious. The presence of fumarate dehydrogenase suggests that OA G20 can be grown on alternative electron acceptor-rich environments (such as fumarate-rich environments). The presence of hydrogenases is strongly dependent on the presence of hydrogen as a donor in the OA G20 growth environment. Therefore, except the conditional genes (dde_1789, thioredoxin disulfide reductase, peroxiredoxin, formate dehydrogenase, and hydrogenases), all the enriched genes mentioned in this study were identified and proposed as essential genes when sulfate and lactate are acting as electron acceptor, and donor, respectively. These genes could be validated using knockout studies in the future.

**Figure 8:**
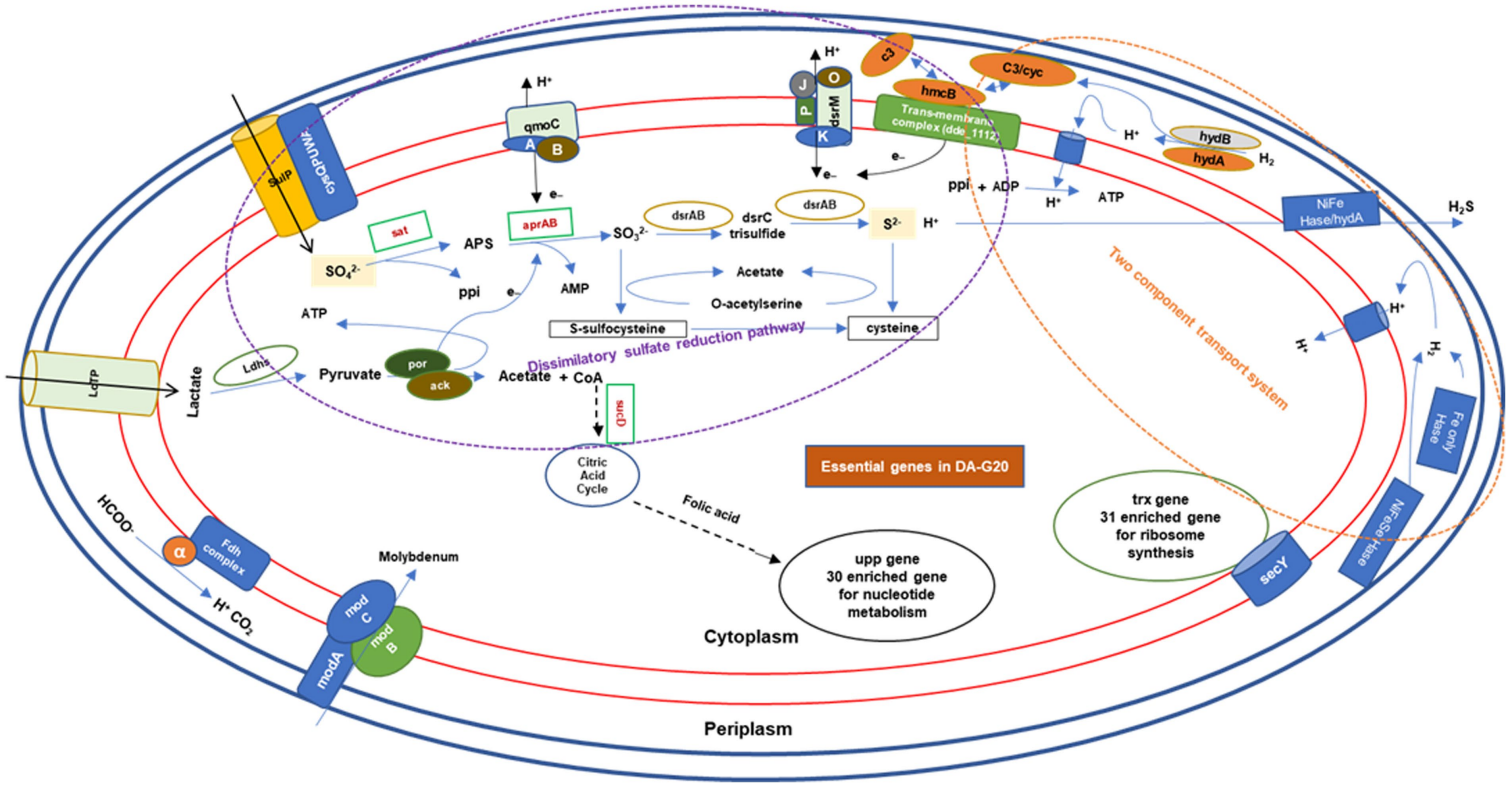
The mechanism of essential genes towards OA G20 survival. AMP, adenosine monophosphate; ATP, adenosine triphosphate; ADP, adenosine diphosphate; LDH, lactate dehydrogenase; LdtP, L,D-transpeptidase; SulP, sulfate permease; por, pyruvate:ferredoxin oxidoreductase; ack, acetate kinase; CoA, CoenzymeA; and ppi, inorganic phosphate.

The Figure 8 explains the involvement of genes in essential mechanisms and pathways for survival of the bacteria. It describes clustering and enrichment of most important group of genes for substrate utilization and sulfate reduction and transmembrane complexes and regulation of these mechanisms (shown in purple dotted circle). Figure showed that *suc*D gene is a connecting link between lactate utilization and citric acid cycle, followed by folic acid which proceed to nucleotide metabolism (30 enriched genes circled in black). Interestingly, the *sat* gene was also observed to be the interlinkage between the dissimilatory sulfate reduction pathway and other three pathways, namely, cysteine and methionine metabolism, selenocompound metabolism, and secondary metabolite synthesis pathway. Genes for two-component transport system (circled in orange dotted lines) involved in hydrogen metabolism and proton generation, transport, and ATP generation. Another important gene *trx*, which networked with other 31 genes involved for ribosome synthesis. There is another set of genes for molybdenum transport, which used as a cofactor by most of the SRBs. With above analysis we tried to predict the essential genes for OA G20.

## 4. Conclusion

The information and knowledge of a set of essential genes in any group of microbes or in an individual bacterium is important to understand the ability in term of growth, management of stresses and survivability. In this study, we have used different approaches (manual and machine learning) for a systematic investigation of “essential genes” in sulfate reducing bacterium OA G20. The manual text mining, PPI network analysis and pangenome analysis were employed to identify the essential gene-set in OA G20. With text-mined 20 genes, we were able to generate clusters of 116 genes by StringDB PPI interactions and further categorized them in gene families based on pangenome analysis. We found 81 genes belong to the persistent category which reveals their conserved distribution across the *Desulfovibrio* genomes and only 25 genes in other categories. These results shed light on 81 genes out of 3,221 genes (coding proteins) of OA G20 to be considered as utmost crucial for organism and therefore could be categorized as “essential genes” in OA G20. However, from the above observations we can conclude that the genes within the shell category depends on the environmental factors of a cell, and that OA G20 can grow under numerous electron donors, but sulfate as the only electron acceptor.

## 5. Future Studies

The proposed framework leveraging diverse tools focus on the expert informed computational analysis principle to discover gene sets of interest from a bio-problem. Improvement will follow for example, our approach towards the investigation of gene essentiality in OA G20 can be validated using molecular-based strategies such as gene knockout or gene silencing with the aid of CRISPR. This will add the experimental validation to our computational validation. Moreover, our priority for the selection of genes for molecular strategies will be focused on those set of genes which showed greater interaction and are key linkers between two pathways. Secondly, we will be focusing on the genes which are involved in essential pathways and are yet unannotated.

## Data availability statement

The original contributions presented in the study are included in the article/Supplementary material, further inquiries can be directed to the corresponding authors.

## Author contributions

PS, RSa, and EG: conceptualization. PS, RSi, RSa, and EG: methodology and investigation. SR, AB, and MA: software. PS, SR, and RSi: validation. PS, PT, AT, RSa, and EG: formal analysis. RSa and EG: resources, supervision, project administration, and funding acquisition. SR, AT, AB, and MA: data curation. PS: writing—original draft preparation. PS, SR, RSi, AT, CL, RSa, PT, and EG: writing—review and editing. PS, SR, MA, PT, and AT: visualization. All authors contributed to the article and approved the submitted version.

## Funding

We gratefully acknowledge support from the National Science Foundation (Awards #1736255, #1849206, and #1920954).

## Conflict of interest

The authors declare that the research was conducted in the absence of any commercial or financial relationships that could be construed as a potential conflict of interest.

## Publisher’s note

All claims expressed in this article are solely those of the authors and do not necessarily represent those of their affiliated organizations, or those of the publisher, the editors and the reviewers. Any product that may be evaluated in this article, or claim that may be made by its manufacturer, is not guaranteed or endorsed by the publisher.
